# Exploring Teacher Reflection in the English as a Foreign Language Context: Testing Factor Structure and Measurement Invariance

**DOI:** 10.3389/fpsyg.2021.828901

**Published:** 2022-02-10

**Authors:** Xing Xiaojing, Ebrahim Badakhshan, Jalil Fathi

**Affiliations:** ^1^College of Teacher Education, University of the Cordilleras, Baguio, Philippines; ^2^Linguistics Department, University of Kurdistan, Sanandaj, Iran; ^3^Department of Applied Linguistics, University of Kurdistan, Sanandaj, Iran

**Keywords:** EFL (English as a foreign language), teacher reflection, factor structure, measurement invariance, confirmatory factor analysis

## Abstract

The current study aimed to verify the multidimensional factor structure of teacher reflection and to examine the psychometric properties of a widely used teacher reflection scale using a large-scale representative dataset of 1,611 practicing Iranian English as a Foreign Language (EFL) teachers. Furthermore, the measurement invariance of the hypothesized, *a priori* six-factor model of teacher reflection as measured by the adapted scale was assessed across gender and educational degree in M*plus* program. In addition, the differences in latent factor means of the same groups were examined. The result of confirmatory factor analysis revealed that teacher reflection was a multidimensional construct, encompassing six underlying factors. Overall, the adapted teacher reflection scale based on the 6-factor model showed an overall good fit. The results also indicated metric and scalar invariance which manifests that the factors underlying the adapted scale had an identical theoretical structure across educational degree/gender groups. Finally, there were significant factor mean differences in reflection components across gender and educational degree groups. A discussion of the results and their implications ensue.

## Introduction

As positive psychology (Seligman and Csikszentmihalyi, 2000) gained momentum in educational fields, second language (L2) researchers have legitimized the investigation of positive emotions in the field of L2 learning (Elahi Shirvan et al., 2021; Wang et al., 2021). L2 teachers should be equipped with practical techniques and strategies to be able to dynamically reshape and refine their language teaching knowledge in order to meet different needs of their L2 learners (Farrell, 2007; Hall, 2018; Greenier et al., 2021). Teacher reflection is argued to be one of these practical techniques helping teachers continually develop, enhance, and reshape their teaching practice in the world of L2 classroom (Farrell, 2015; Fat’hi and Behzadpour, 2011; Gkonou and Miller, 2021; Hahl, 2021). In L2 teaching literature, reflective practice has evolved as the approach in which practitioners actively gather information regarding their instructional perceptions and activities and then think over the information to make proper pedagogic choices (Farrell, 2007, 2015; Murphy, 2013).

In spite of the numerous references made to teacher reflection in English Language Teaching (ELT) as a critical education movement, which should be favored in development programs of teachers (Jay and Johnson, 2002; Farrell, 2006, 2007, 2015), few empirical studies have investigated the efficacy and viability of reflection for ELT practitioners. In spite of the fact that teacher reflection is context-sensitive (Soodmand Afshar and Farahani, 2018), this construct still requires further exploration in L2 contexts Farrell and Baecher, 2017; Moradkhani and Shirazizadeh, 2017). Although an array of studies have investigated teacher reflection in ELT contexts (Fathi et al., 2021), few studies have ever investigated the psychometric properties of the scales measuring reflective teaching. More specifically, the widely used reflection scale in second language (L2) pedagogy is that of Akbari et al. (2010), which proposes a six-factor model for reflective teaching based on which a 29-item inventory for measuring reflection was developed and validated. They proposed a six-factor model of reflective teaching comprising practical, cognitive, meta-cognitive, affective, critical and moral reflection. Nevertheless, in the final version of their developed inventory, the moral factor of teacher reflection did not survive the confirmatory factor analysis (CFA) stage, thereby reducing the final scale to a five-factor model. But as far as L2 teacher reflection is concerned, moral reflection is an important dimension which has attracted a heightened attention (Valli, 1990; Hansen, 1998; Farrell, 2015). Given the significant role of moral dimension of teacher reflection (Hansen, 1998; Akbari et al., 2010) and also the necessity of replication studies regarding instrument validation, further studies are deemed legitimate to quantify L2 teacher reflection more effectively and to refine the factor structure of the proposed model more appropriately.

Additionally, validation of assessment instruments has gathered momentum in L2 research over the last recent decades (Dörnyei, 2010). A part of validity investigation requires comparing the groups on an underlying factor in order to verify that the scale is perceived and understood identically for each group (Byrne and Watkins, 2003; Dimitrov, 2010). This part of validation process is conceptualized as testing for *factorial invariance* (Byrne, 2004; Wu and Zumbo, 2007), which is of great value in marshaling evidence regarding particular dimensions of construct validity (Dimitrov, 2010). To make valid model comparisons by group, it is necessary to evaluate to what extent the measurement structure of the model is invariant across different groups (Cheung and Rensvold, 2002). In spite of its undeniable significance (Putnick and Bornstein, 2016), testing for factorial invariance across groups has not been systematically addressed in applied linguistics.

Therefore, the purpose of the present study was to empirically confirm the multidimensional factor structure of previously hypothesized model of L2 teacher reflection (Akbari et al., 2010) and to test the psychometric properties of the scale (Akbari et al., 2010) and its measurement invariance across gender and educational degree. From a technical and psychometric point of view, the current study tapped into the *structural* and *generalizability* aspects of the unitary concept of construct validity (Messick, 1995) through testing for model fit and testing for measurement invariance respectively. Finally, the differences in latent factor means of the gender/educational degree groups were examined.

### Teacher Reflection

Within the accumulated body of the literature dealing with teacher reflection, some educationalists and scholars believe that reflection is the key element of successful instruction and effective teachers are engaged in systematic examination of or critical reflection on their beliefs and practices so that they can enhance their own performance in the classroom (Jay and Johnson, 2002; Farrell, 2006, 2007, [Bibr B17][Bibr B40]; Gkonou and Miller, 2021). Numerous scholars have also tried to provide the comprehensive definition or typology for reflection and reflective practice (Van Manen, 1977; Schon, 1987; Valli, 1990; Jay and Johnson, 2002).

Jay and Johnson (2002, p. 76) suggest “reflection is a process, both individual and collaborative, involving experience and uncertainty. It is comprised of identifying questions and key elements of a matter that has emerged as significant, then taking one’s thoughts into dialogue with oneself and with others.” Van Manen (1977) also viewed reflective teaching as a composite of three components of technical rationality, practical reflection, and critical reflection. Technical rationality includes lower levels of reflection and addresses technical application of the knowledge and skills in the classroom. Practical reflection emphasizes reviewing and analyzing the conceptions underlying practice. And finally at the highest levels of reflection, critical reflection centers on the moral and ethical issues such as justice and equity that affect the practice of teaching.

In another taxonomy, Valli (1990) views reflective practice as consisting of five key steps forming a hierarchy. In Valli’s hierarchy, technical reflection is concerned with checking one’s teaching against other criteria such as those for research and standards. Reflection in/on action deals with problem solving in a particular classroom context. Deliberative reflection encompasses having different points of view and research to better understand the various issues in teaching. Personalistic reflection emphasizes teachers’ personal development and relations; it involves considering different viewpoints including one’s own view and those of others to gain perspective on a given situation. Valli’s critical reflection is related to the ethical, moral, political, and social issues.

Schon (1983) characterizes two forms of reflection: reflection-in-action and reflection-on-action. Reflection-in-action represents practitioners’ active thinking and understandings at the moment of teaching. This online process pertains to teachers’ interpretations of and reactions to what happens at the moment of instruction. Reflection on action, conversely, is posteriori and transpires after the act of teaching. This type of reflection is realized in teachers’ post-action deliberations over what occurred in the classroom from recollecting instruction.

Jay and Johnson (2002) introduced a typological framework for reflective practice encompassing three dimensions of reflective thought: descriptive, comparative, and critical. From their perspective, the first dimension of reflective thought is descriptive reflection that involves the intellectual process of “setting the problem”. During this stage of the reflective activity, “problem setting” and “problem identification” take place. “Problem” here refers to any confusing or troublesome situation or phenomenon that practitioners may face in their educational activities. The comparative dimension of this reflection typology “involves thinking about the matter for reflection from a number of different frames or perspectives” (Jay and Johnson, 2002, p. 78). Within the comparative stage of the reflection process, a practitioner tries to expand his/her perspectives on the problem by analyzing it from different angles and developing a new frame of reference. Critical reflection constitutes the third dimension of the reflection typology. During the critical reflection as the third dimension of the reflection typology, the practitioner makes a judgment or a choice from among different alternatives to the problem. This level of reflection also involves considering the historical, socio-political, and moral context of education and schooling.

One recent framework developed for reflective practice has been introduced by Farrell (2015). This framework constitutes five levels or stages of reflection: philosophy, principles, theory, practice, and beyond practice. Subscribing to a philosophy of practice posits that every behavior or action has a rationale behind it even if the practitioner does not express it. In order to be able to reflect on his own underlying philosophy, a practitioner needs to acquire an inner knowledge of his self that can be accessible through reflection on the various issues such as his heritage, ethnicity, religion, socioeconomic background, family and personal values which have accumulated over years to shape and impact who the person is as a language teacher. At the level of principles, the teacher reflects on his assumptions, beliefs, and conceptions of learning and teaching. At the third level of reflection (theory), which is affected by reflections on philosophy and principles, the practitioner intends to create his theory of practice. The various elements of this level of reflection include aspects of a teacher’s planning and the different activities and methods teachers employ. Reflection at the level of practice includes reflecting on the more tangible and immediate behaviors of teaching and thinking over what actually takes place in his class. And finally, the “beyond practice” reflection refers to the “critical reflection” that “entails exploring and examining the moral, political and social issues that impact a teacher’s practice both inside and outside the classroom” (p. 8).

In a study more relevant to the purpose of the present study, Akbari et al. (2010) proposed a multi-dimensional model for the L2 teacher reflection. Their hypothesized six-factor model of L2 teacher reflection was developed after the examination of experts’ opinion and a comprehensive review of the related literature. Taking the hypothesized model as the point of departure, the authors developed and validated a 29-item reflection inventory to measure and quantify reflection among English language teachers. They tried to create a theoretically-grounded instrument which captured the multiple dimensions of the L2 teacher reflection. However, the final version of the developed inventory lacked the morality dimension as its underlying factor. In other words, since the moral reflection did not survive the CFA stage, it was removed from the model, reducing the model to a five-factor model.

The six-component hypothesized model which was believed to encompass the domains of L2 reflection for teachers included six elements: practical, cognitive, learner (affective), meta-cognitive, critical and moral. The *practical* element of teacher reflection refers to practitioners’ reflective activities and their employed tools for reflection. Diaries, lesson reports, questionnaires, audio/video recordings, observation, action research, teaching portfolios, group discussions, and analysis of critical incidents are among the various tools employed by the teachers for the reflective practice. The *cognitive* element of reflection pertains to teachers’ attempts and initiatives toward professional development. The cognitive reflection includes practitioners’ self-initiated activities like conducting action research, attending conferences and workshops, and studying the professional literature and publications in the domain of ELT. The *affective* reflection is concerned with teachers’ reflections about students, and the ways they learn. The learner (or affective) element also addresses teachers’ reflection on their students’ emotional make-up and their emotional responses and/or reactions to what transpires in the classroom. The *meta-cognitive* element of the reflective instrument deals with teachers and their instances of reflection on their own beliefs and personality, and the way they define their practice. This component also addresses teachers’ reflection on their own emotional make-up and how teachers’ personal characteristics affect their professional practices. The *critical* element of reflection relates to teachers’ reflection on the socio-political aspects of pedagogy. More specifically, this dimension deals with teachers’ reflections about the political significance of their practice. The issues and topics relating to race, gender and social class, and teachers’ initiatives toward student empowerment also fall within this domain of the reflective practice. And finally the *moral* element focuses on teachers’ reflecting on moral issues such as empathy, equality and values. This element deals with the moral aspects of personal features and how people view and treat others.

### The Significance of Measurement Invariance

In line with the substantial shift of attention and orientation toward considering teachers as the key players in the classroom both in mainstream education (Sanders, 1998, 2000) and in ELT (Freeman and Johnson, 1998), a burgeoning research base has been devoted to empirical studies investigating the teacher variables such as sense of efficacy (Bandura, 1997), reflection (Schon, 1987; Pultorak, 1993), pedagogical knowledge (Gatbonton, 2008), burnout (Maslach and Jackson, 1981) and teaching styles (Miglietti and Strange, 1998). Moreover, a significant number of studies exploring the teacher variables have employed validated scales or instruments in quantitative studies. Since teacher reflection might be an alluring construct as far as the teacher-related variables are concerned (Farrell, 2007; Gkonou and Miller, 2021), it seems necessary to make efforts in enhancing the psychometric properties of the assessment instrument and to consider measurement error by computing the measurement errors in validation process. One part of the validation process, as discussed above, is to test for measurement invariance. Without measurement invariance evidence, group comparisons of teacher variables might be substantially biased (Byrne, 1998; Byrne and Stewart, 2006).

Measurement invariance typically pertains to the extent to which an item in a survey or instrument is being understood identically across groups (Byrne and Watkins, 2003). Many researchers in behavioral sciences seek to explore if a scale has the identical psychometric properties across different groups. Measurement invariance is of great significance as far as group comparison is concerned. The key point to be dealt with before making comparisons across groups is if the construct is interpreted in the same way for each group. When measurement invariance could not be substantiated, then the findings of between-group differences are not clearly interpreted. In such cases, non-uniform psychometric responses to the scale items are likely to distort the measurement of the construct of interest. Measurement invariance is normally tested at a series of levels. Widaman and Resie (1997) have proposed techniques for evaluating a number of hierarchical models to investigate measurement invariance.

The first level of measurement invariance is testing for configural invariance which shows the invariance of pattern of free and fixed model parameters across groups. This level of invariance needs that the same item be corresponded with the same factor in each group; nevertheless, the factor loadings might vary across groups. Configural invariance shows that similar, but not the same, latent factors have been assessed in the groups (Widaman and Resie, 1997). The second level of measurement invariance is concerned with testing for the factor loadings. Factor loadings reveal the power of the linear correlation between each construct and its related items (Jöreskog and Sörbom, 1999). If the factor loading of each item on the underlying factor is identical across groups, it indicates that the underlying factor is identical in terms of unit or interval.

The third level of invariance aims to test for the invariance at the intercept level. Intercepts refer to the origin of the scale. If intercept invariance is achieved, it suggests that scores of the heterogeneous groups possess both the same unit of measurement and the same origin. We test for intercept invariance when we intend to compare latent mean differences across groups (Bollen, 1989). Finally, the fourth level of measurement invariance is tested at the residual invariance level. In case this level of invariance is achieved, the differences between groups on the items are only attributed to group differences on the common factors. Nevertheless, there is a general agreement that it is not necessary to establish such invariance across groups on these parameters (Dimitrov, 2010).

### The Current Study

**FIGURE 1:**
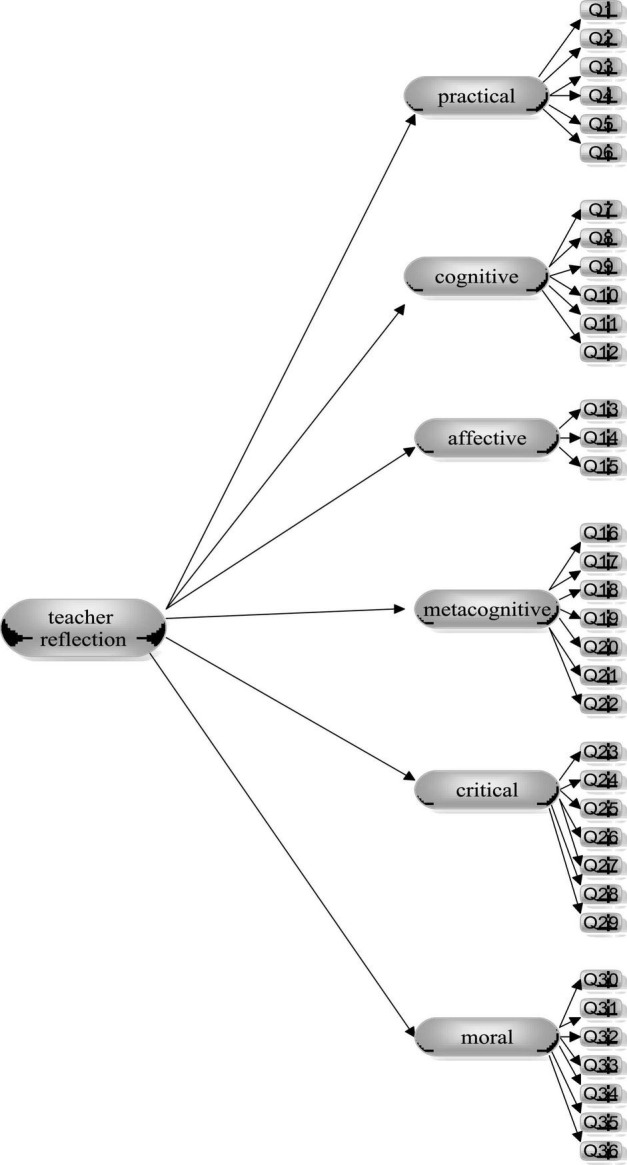
Hypothesized model of teacher reflection.

The current study seeks to accomplish three objectives. First, the dimensional structure of an *a priori* six-factor solution of teacher reflection and the psychometric properties of a slightly adapted teacher reflection scale were examined. Second, the measurement invariance of the hypothesized, *a priori* six-factor model of teacher reflection (see Figure 1) as measured by the adapted scale was assessed across gender and educational degree. Third, the latent variable mean differences across groups were compared. In so doing, the following research questions were addressed:

1. Does the construct of L2 teacher reflection show a multidimensional factor structure?
2. Does the multidimensional factor model of L2 teacher reflection display measurement invariance separately by gender and educational degree?
3. Are there gender and educational degree group differences in L2 teacher reflection and is the pattern of difference the same across the reflection components?

## Materials and Methods

### Participants and Procedure

A sizable number of in-service Iranian English teachers with various ages, genders, instructional backgrounds, degrees, and experience were recruited as the participants. The slightly adapted teacher reflection scale was administered to the total number of approximately 2,300 practicing (in-service) English instructors at various institutes, schools, and centers of higher education in geographically various regions/provinces (i.e., east. west, north, and south) in Iran. Both online and face-to-face versions of the survey were employed for the data collection. Each administered questionnaire took approximately 20 min to be completed. Among the 2,300 administered questionnaires, 1,704 questionnaires were filled out by the participants and given back to the researchers (a response rate of 74%). After careful examination of the filled instruments, 93 questionnaires were excluded as they were either partially answered or heedlessly filled out. This provided the researchers with the total number of 1,611 filled instruments. The data collected from the participants (*n* = 1,611) indicated that their age varied from 19 to 49 with the average age of 27.16. The mean teaching experience was 9.84 years (ranging from 1 to 38 years). They were both male (41.77%) and female (58.35%), with 34 individuals (2.11%) as unspecified. Concerning the educational degree, 961 teachers (60.65%) had bachelor degree (BA), 559 teachers (34.69%) had master degree (MA) and 22 teachers (1.36%) held Ph.D. in English majors. The 42 teachers (2.60%) labeled as *others* (see Table 1) were the teachers who had diploma, associate diploma in English majors or their educational degree was Non-English-related. Finally, 27 teachers (1.67%) were unspecified in terms of the educational degree. Table 1 indicates teachers’ demographic information.

**TABLE 1:** Teacher demographic information.

	Sample 1 (*n* = 785)		Sample 2 (*n* = 826)		Total sample 1 (*n* = 1611)
	*N*	%	*N*	%	*N* (%)
**Gender**					
Male	342	43.56	331	40.07	673 (41.77)
Female	422	53.75	482	58.35	904 (56.11)
Unspecified	21	2.67	13	1.57	34 (2.11)
**Degree**					
Bachelor (BA)	467	59.49	494	59.80	961 (60.65)
Master (MA)	280	35.66	279	33.77	559 (34.69)
Ph.D.	8	1.01	14	1.69	22 (1.36)
Others	19	2.42	23	2.78	42 (2.60)
Unspecified	11	1.40	16	1.93	27 (1.67)

For the purpose of the study, the entire sample (*n* = 1611) was randomly divided in sample 1 (*n* = 785) and sample 2 (*n* = 826) by performing random split through the SAMPLE command in SPSS.

### Instrumentation

#### English Language Teacher Reflective Inventory

The only existing English language teacher reflection scale is the instrument called the English Language Teacher Reflective Inventory (ELTRI) which was developed by Akbari et al. (2010) based on reflection elements. Their hypothesized model of L2 teacher reflection was a six-component model encompassing practical, cognitive, meta-cognitive, affective, critical and moral reflection. Nevertheless, CFA did not reveal a satisfactory fit for the moral factor, resulting in the development of a five-factor inventory of the teacher reflection as the final version of the validated instrument. Therefore, ELTRI is a 29-item self-report inventory based on the five underlying factors including practical (6 items), cognitive (6 items), learner (affective) (3 items), meta-cognitive (7 items), and critical (7 items) elements. In ELTRI, the practical component pertains to issues like keeping diaries, discussing with colleagues, and portfolio instruction. Cognitive component is concerned with the directed attempts for professional growth such as reading journals and books. The learner (affective) component deals with knowing about learner’s affective and cognitive state. Meta-cognitive factor centers on teachers’ consciousness of their personality features. Finally, socio-political dimensions of pedagogy are the related to the critical component of reflective teaching.

However, given the paramount significance of the moral element as an important element of teacher reflection (Valli, 1990; Hansen, 1998; Farrell, 2015), the original version of ELTRI was slightly adapted for the purpose of the present study. In so doing, slight modifications were made to the original scale, resulting in the adapted scale (adapted ELTRI) which included the 29 items previously developed in the original scale accompanied by 7 new items representing the moral reflection. Among the seven newly added items, two items were borrowed from the initial 42-item instrument developed by Akbari et al. (2010). Development and selection of the new items (7 items) were drawn from the review of theoretical underpinning of the moral reflection in the literature. Moreover, some experts in the area of teacher reflection were consulted in the development of these new items.

The final set of 36 items of the adapted scale is presented in the first column of Table 2. The teachers were supposed to rate the extent to which they agreed with each statement using a 5-point Likert scale (1 = Never; 2 = Rarely; 3 = Sometimes; 4 = Often; and 5 = Always).

**TABLE 2:** Adapted ELTRI items: standardized factor loadings of six-factor model (36 items) in sample 1 and sample 2.

Items	Standardized factor loadings	R^2^
1. I have a file where I keep my accounts of my teaching for reviewing purposes. **(P)**	54/55	30/31
2. I talk about my classroom experiences with my colleagues and seek their advice/feedback. **(P)**	63/69	41/48
3. After each lesson, I write about the accomplishments/failures of that lesson or I talk about the lesson to a colleague. (P)*		
4. I discuss practical/theoretical issues with my colleagues. **(P)**	71/71	51/50
5. I observe other teachers’ classrooms to learn about their efficient practices. **(P)**	58/52	33/26
6. I ask my peers to observe my teaching and comment on my teaching performance. **(P)**	43/46	19/21
7. I read books/articles related to effective teaching to improve my classroom performance. **(Co)**	56/53	30/27
8. I participate in workshops/conferences related to teaching/learning issue. **(Co)**	64/59	41/34
9. I think of writing articles based on my classroom experiences. (Co)*		
10. I look at journal articles or search the internet to see what the recent developments in my profession are. **(Co)**	67/76	43/58
11. I carry out small scale research activities in my classes to become better informed of learning/teaching processes. **(Co)**	57/51	33/26
12. I think of classroom events as potential research topics and think of finding a method for investigating them. **(Co)**	69/73	48/53
13. I talk to my students to learn about their learning styles and preferences. **(A)**	44/49	20/24
14. I talk to my students to learn about their family backgrounds, hobbies, interests and abilities. **(A)**	65/65	42/42
15. I ask my students whether they like a teaching task or not. **(A)**	58/54	33/30
16. As a teacher, I think about my teaching philosophy and the way it is affecting my teaching. **(Me)**	78/75	60/56
17. I think of the ways my biography or my background affects the way I define myself as a teacher. (Me)*		
18. I think of the meaning or significance of my job as a teacher. **(Me)**	62/66	38/42
19. I try to find out which aspects of my teaching provide me with a sense of satisfaction. **(Me)**	71/74	51/57
20. I think about my strengths and weaknesses as a teacher. **(Me)**	48/43	23/19
21. I think of the positive/negative role models I have had as a student and the way they have affected me in my practice. **(Me)**	58/57	34/32
22. I think of inconsistencies and contradictions that occur in my classroom practice. **(Me)**	73/77	54/59
23. I think about instances of social injustice in my own surroundings and try to discuss them in my classes. **(Cr)**	63/67	40/45
24. I think of ways to enable my students to change their social lives in fighting poverty, discrimination, and gender bias. **(Cr)**	53/49	28/24
25. In my teaching, I include less-discussed topics, such as old age, AIDS, discrimination against women and minorities, and poverty. **(Cr)**	75/79	58/61
26. I think about the political aspects of my teaching and the way I may affect my students’ political views. **(Cr)**	70/72	49/51
27. I think of ways through which I can promote tolerance and democracy in my classes and in the society in general. (Cr)*		
28. I think about the ways gender, social class, and race influence my students’ achievements. **(Cr)**	58/55	33/30
29. I think of outside social events that can influence my teaching inside the class. **(Cr)**	48/53	23/28
30. I consider my teaching as an opportunity to express sympathy and care to others. (Mo)*		
31. I teach my students the moral principles they need to live in society. (Mo)*		
32. I try to act as a model of moral standards and values for my students. **(Mo)**	64/69	41/47
33. I believe in moral and ethical issues and try to show such beliefs in my classroom practice. **(Mo)**	67/67	45/44
34. I provide equal opportunities for all my students in the class regardless of their capabilities. **(Mo)**	55/58	31/35
35. I have a clear set of general class rules and what constitutes acceptable behavior for my students to follow. **(Mo)**	79/72	62/53
36. I try to encourage my students to follow the moral principles in their life. **(Mo)**	61/52	38/27

The assignment of each item to factor has been presented in parentheses following the item. P, Practical; Co, Cognitive; A, Affective; Me, Metacognitive; Cr, Critical; and Mo, Moral. *Items removed from the adjusted model. Standardized factor loadings for sample 1 has been presented on the left size of the slash, and standardized factor loadings for sample 2 appear on the right size of the slash.

The reliability of the adapted ELTRI was assessed by calculating the internal consistency coefficients for each of the components for the total sample (*N* = 1611), for sample 1 (*n* = 785) and for sample 2 (*n* = 826). Cronbach’s α coefficients for the practical component ranged from 0.80 for sample 1 to 0.88 for sample 2 and 0.91 in the total sample. For the cognitive reflection, Cronbach’s α coefficients ranged from 0.92 for sample 1 to 0.87 for sample 2 and 0.83 in the total sample. For the affective factor, the values were 0.88 for sample 1 to 0.86 for sample 2 and 0.84 in the total sample. Alpha values for the metacognitive reflection also ranged from 0.79 for sample 1 to 0.80 for sample 2 and 0.78 in the total sample. Similarly, they ranged from 0.78 for sample 1 to 0.86 for sample 2 and 0.81 in the total sample for the critical reflection. And Cronbach’s α coefficients for the moral component were 0.84 for sample 1 to 0.86 for sample 2 and 0.81 in the total sample. Cronbach’s α values for the whole adapted ELTRI ranged from 0.88 for sample 1 to 0.92 for sample 2 and 0.83 in the total sample, verifying the relatively high reliability indices for the adapted ELTRI and its underlying subscales. Table 3 shows the internal consistency measures for the adapted ELTRI and its sub-scales in sample 1, sample 2 and the total sample.

**TABLE 3:** Cronbach’s alpha coefficients of the adapted ELTRI and its subscales in sample 1, sample 2 and the total sample.

	Sample 1 (*n* = 785)	Sample 2 (*n* = 826)	Total sample (*n* = 1611)
Practical	0.80	0.88	0.91
Cognitive	0.92	0.87	0.83
Affective	0.88	0.86	0.84
Metacognitive	0.79	0.80	0.78
Critical	0.78	0.86	0.82
Moral	0.84	0.86	0.81
Whole scale	0.88	0.92	0.83

### Statistical Analyses

To analyze the data and to examine the adequacy of the models, confirmatory factor analyses were performed, employing the program M*plus* 7.11 (Muthén and Muthén, 2012) in some steps. The analyses were performed with the MLR estimator in Mplus program.

In the first step, to evaluate the proposed six-factor model of teacher reflection including the 36 items, a CFA was performed to the collected data of sample 1. Covariance structures with the maximum likelihood (ML) method were used. To evaluate goodness-of-fit of the hypothesized model of reflection, the Satorra and Bentler (1988) scaled chi-square statistic (SBχ2) and other fit indices were used. Since the multivariate normality is not guaranteed in the current sample, the SBχ2 that considers the non-normality of the data is recommended (Satorra and Bentler, 1994). Moreover, Root Mean Square Error of Approximation (RMSEA), Standardized Root Mean Square Residual (SRMR), and the Comparative Fit Index (CFI) were also investigated. Statistically, the model is viewed as acceptable when CFI ≥ 0.90 and good when CFI ≥ 0.95 (Bentler, 1992; Hu and Bentler, 1999). In addition, for a good fit, SRMR should not exceed 0.08 (Hu and Bentler, 1999). Furthermore, RMSEA values ≤ 0.06 are viewed to be indicator of good fit, ≤ 0.08 of fair fit, between 0.08 and 0.10 of mediocre fit and > 0.10 of poor fit (Hu and Bentler, 1999). To identify the items causing misfit, standardized residuals and modification indices were employed. The items causing misfit were either reviewed or discarded. In the second step, a CFA was run once again with the adjusted model using the same dataset of sample 1, and in the third step, another CFA was performed to cross-validate the previous adjusted model using the dataset of sample 2.

Then, following the general suggestions by Widaman and Resie (1997) and employing a multistage procedure known as *forward* approach (Dimitrov, 2010), a hierarchical array of the nested factor models was fit in the analyses to assess measurement invariance. The forward (or sequential constraint imposition) approach to testing for invariance across groups is contingent on one of difference chi square tests (Δχ2 or ΔSBχ2) between two nested models: a constrained model and unconstrained model for particular parameters (e.g., factor loadings and intercepts). Invariance of the parameters being tested is approved when the difference test (Δχ2 or ΔSBχ2) is not statistically significant at a pre-specified level of significance (e.g., 0.05) (Dimitrov, 2010). Such investigation starts with the most unconstrained structure representing entire absence of invariance and then limitations for the equality of the particular variables across groups are imposed, thereby creating the nested models which are tested against each other employing the difference test.

Given the above discussion, a baseline model was established in each group followed by tests for equivalence across groups at a number of increasingly more constrained levels. The baseline model is the most parsimonious but the most meaningful and best-fitting model to the data for a group (Byrne, 2004). Every pair of the models was nested in the analysis since a number of parameters were constrained to be equal across groups in the more constrained model. In order to test measurement invariance, one of the factor loadings was fixed to 1 and the constraints were added sequentially.

In Model 0 which was intended to test configural invariance (Horn et al., 1983), the number of factors and pattern of fixed and free factor loadings were constrained to be the same across the gender and educational degree groups. Yet, different estimators for the corresponding parameters were allowed. In configural invariance investigation, a baseline model is identified and estimated separately for each group. In fact, this unconstrained multi-group model was considered as the baseline model against which the fitness of more constrained models were estimated.

Model 1 aimed at testing for the factor loading invariance which is referred to as *metric invariance*. In this model, all factor loadings were constrained to be the same across groups. Model 2 was used to test for intercept of the observed variables invariance. In such a measurement invariance, which is also called *scalar invariance*, the intercepts of the observed variables, as well as the constraints on the factor loadings of the latent variables were constrained to be equal. Model 3 tests for the intercept of the latent factorial invariance. To test for this level of invariance, the factor loadings, the intercepts of the observed variables, and the means of the factors were constrained to be equal across groups. In case this level of invariance is obtained, it reveals that the factor loadings and the intercepts are identical across groups.

To examine the fit of the models, ΔSBχ2 (Satorra and Bentler, 1988) statistic was used. If ΔSBχ2 statistic is significant, it indicates that the constraint imposed in the more constrained model is not identical across groups. Conversely, in case ΔSBχ2 statistic fails to be significant, it indicates that the equality constraints have been valid and the constrained model can be accepted. Since ΔSBχ2 statistic is sensitive to large sample sizes and non-normality of the data (Tomarken and Waller, 2003), it is suggested to employ other fit indices for model evaluation (Marsh et al., 1997; Cheung and Rensvold, 2002). The recommendations made by Cheung and Rensvold (2002) were taken into account in this research. Due to the sensitivity of χ2 or SBχ2, Cheung and Rensvold (2002) suggested that researchers use ΔCFI accompanied by χ2 or SBχ2 results on testing for invariance. They proposed that ΔCFI should be smaller than 0.01 (Dimitrov, 2010). As a result, both ΔSBχ2 and ΔCFI were employed in assessing the model fit. If there is a discrepancy between these two statistics, the changes in CFI were relied on because of the significant sample size in this research (Chen et al., 2005).

Ultimately, to investigate the group differences in L2 teacher reflection and to see if the pattern of difference is the same across groups, the mean differences on the reflection factors by group were tested. In so doing, one group was considered the reference group for which the factor means were set to zero while the comparison groups’ factor means were estimated to be free. These freely estimated latent means, as a result, indicate the difference between the factor means of the two groups. And to examine distinctions between the two groups’ latent means, a z statistic was used (Sörbom, 1978; Aiken et al., 1994).

## Results

### Testing the Factor Structure

#### Step 1: Hypothesized Six-Factor Model in Sample 1

In the first step, goodness-of-fit indices for the hypothesized model (see *Model 1* in Table 4) of L2 reflection (encompassing six underlying factors of practical, cognitive, meta-cognitive, affective, critical and moral reflection) as measured by the adapted 36-item scale were tested based on the collected data in sample 1. The fit indices of the model were not satisfactory, SBχ2 (342) = 572.86, *p* < 0.05; CFI = 0.851, RMSEA = 0.062, and SRMR = 0.084. Further examination indicated that the values of the standardized residuals were very big for items 3, 9, 17, 27, and 30 with accordingly small standardized factor loadings. Moreover, the modification indices revealed a significant covariance between item 31 and item 36 (both from the moral factor). The scrutiny of the content of these two items revealed greatly identical wording.

**TABLE 4:** Fit indices of the hypothesized and adjusted model for in sample 1 and sample 2.

Model	SBχ 2	df	RMSEA	SRMR	CFI	Model comparison	Δ SBχ 2	Δ df
*Model 1*	572.86	342	0.062	0.084	0.851	–	–	–
*Model 2*	3792.12	424	0.053	0.058	0.966	–	–	–
*Model 3*	3978.26	431	0.057	0.064	0.962	3 vs. 2	142.53	7

* p < 0.01.

Given these obtained findings, six items (3, practical factor; 9, cognitive factor; 17, metacognitive factor; 27, critical factor; 30 and 31, moral factor) were discarded from the hypothesized model. It should be also noted that items 3, 9, 17, and 27 were the items of the original teacher reflection scale (ELTRI).

#### Step 2: Adjusted Model in Sample 1

In the second step, the fit of the adjusted model (see *Model 2* in Table 4) was examined on the remaining 30 items (i.e., the 25 items from the original ELTRI and 5 newly added items for the morality factor) in sample 1. The goodness-of-fit indices of the model were good (SBχ2 (424) = 3792.12, *p* < 0.05; CFI = 0.966, RMSEA = 0.053, SRMR = 0.058), indicating that the suggested six-factorial structure is in line with the data. Table 2 shows the standardized factor loadings for these 30 items. The six items that were removed from the scale have been marked with asterisk (*) in Table 2, and their factor loadings have not been presented.

#### Step 3: Cross-Validation of the Adjusted Model in Sample 2

The primary purpose of the third step was to cross-validate the adjusted model in another sample (see *Model 3* in Table 4). Therefore, a CFA was carried out to validate the fit of the adjusted model in sample 2. The goodness-of-fit indices of the model for sample 2 were satisfactory and very similar to the fit indices of the model for sample 1 [SBχ2 (431) = 3978.26, *p* < 0.05; CFI = 0.962, RMSEA = 0.057, SRMR = 0.064]. The differences in fit between the adjusted model in sample 1 and the corresponding adjusted model in sample 2 were not statistically significant (ΔSBχ2 = 142.53, df = 7, *p* = 0.14) and ΔCFI value was less than 0.01, the cutoff value suggested by Cheung and Rensvold (2002). According to these outcomes, it can be argued that the CFA of the teachers’ responses in the two samples to the 30-item adapted ELTRI confirmed that the six-factor hypothesized model including practical, cognitive, metacognitive, critical, affective and moral factors were fit the data.

### Testing Measurement Invariance by Gender

#### Configural Invariance (Model 0a)

The configural model was considered as the baseline model. In this model, no equality constraints were imposed between groups, served as the baseline model.

As Table 5 indicates, the fit indices reveal a good fit for the configural model (SBχ2 = 248.54; df = 143; *p* < 0.001; RMSEA = 0.042; SRMR = 0.045; and CFI = 0.976).

**TABLE 5:** Fit indices of the nested models for testing the measurement invariance by gender.

Model	SBχ 2	df	RMSEA	SRMR	CFI	Model comparison	Δ SBχ 2	Δ df
*Model 0a*	248.54	143	0.042	0.045	0.976	–	–	–
*Model 1a*	259.83	151	0.042	0.048	0.972	1a vs. 0a	10.45	8
*Model 2a*	266.56	155	0.042	0.049	0.966	2a vs. 1a	6.33	4
*Model 3a*	313.32	164	0.043	0.051	0.961	3a vs 2a	42.72*	9

*p < 0.01.

#### Invariance of Factor Loadings (Model 1a)

When all the factor loadings were constrained to be equal across both male and female groups, this constrained model provided a good overall fit to the data (SBχ2 = 259.83; df = 151; *p* < 0.001; RMSEA = 0.042; SRMR = 0.048; and CFI = 0.972) the difference in SBχ2 (ΔSBχ2) between Models 0a and 1a was small (ΔSBχ2 = 10.45, df = 8, *p* = 0.23) and the ΔCFI was not more than 0.01. These results suggest that the factor loadings are invariant by gender. More technically, it is argued that *metric invariance* is in place, suggesting the presence of identical correlations between a latent factor and its corresponding indicators (items) in the CFA model.

#### Invariance of Intercepts of Observed Variables (Model 2a)

Apart from the constraints previously imposed on the factor loadings, when the intercepts of the observed variables were constrained to be equal by gender, the fit indices for the constrained model provided a good overall fit (SBχ2 = 266.56; df = 155; *p* < 0.001; RMSEA = 0.042; SRMR = 0.049; and CFI = 0.966). The difference in SBχ2 statistic value between Models 2a and 1a was small and statistically insignificant (ΔSBχ2 = 6.33, df = 4, *p* = 0.36), suggesting no significant difference in the intercepts of the observed variables between female and male teachers.

#### Invariance of Intercepts of Factor (Model 3a)

In testing for this model, factor loadings and the intercepts of the observed variables and the intercepts of factor means were all constrained to be equal by gender. The resulting fit statistics for this constrained model showed a good overall fit to the data (SBχ2 = 313.32; df = 164; *p* < 0.001; RMSEA = 0.043; SRMR = 0.051; and CFI = 0.961). The difference in the SBχ2 statistic between Models 3a and 2a was big and statistically significant (ΔSBχ2 = 42.72, df = 9, *p* < 0.001). Nevertheless, following the recommendation of Cheung and Rensvold (2002), we employed the ΔCFI index to evaluate the difference in the model fit. As seen in Table 5, the CFI index declined less than 0.01 (from 0.966 to 0.961), and this reveals that there are no significant differences between Models 3a and 2a. According to these findings, it seems to be no siginficant difference in the intercepts of the hypothesized factors between male and female teachers and it can be concluded that the intercepts of the hypothesized factors are invariant by gender.

### Testing Measurement Invariance by Educational Degree

To test the measurement invariance of the hypothesized factor structure by educational degree, a number of nested models resembling those conducted for gender (see Table 6) were examined. Since the number of Ph.D. teachers and teachers categorized as “others” was negligible, the overriding focus of this part of the analysis was on the comparison between BA and MA teachers.

**TABLE 6:** Fit indices of the nested models for testing the measurement invariance by educational degree.

Model	SBχ 2	df	RMSEA	SRMR	CFI	Model comparison	Δ SBχ 2	Δ df
*Model 0b*	225.81	143	0.047	0.049	0.959	–	–	–
*Model 1b*	233.79	151	0.047	0.052	0.955	1b vs. 0b	7.31	8
*Model 2b*	241.32	155	.048	0.055	0.949	2b vs. 1b	6.81	4
*Model 3b*	289.16	164	0.048	0.058	0.946	3b vs. 2b	44.48*	9

*p < 0.01.

In testing for measurement invariance across the BA and MA samples of teachers, the fit indices for the configural model (see Model 0b in Table 6) was acceptable. Upon examining the baseline model as the point of departure, the invariance of the factor loadings (Model 1b in Table 6) was assessed. By imposing equality constraint on factor loadings by educational degree, the difference in SBχ2 between the models was not significant. These results indicate that the factor loadings for the BA and MA EFL teachers are equivalent. Then, to test for intercept of latent factorial invariance, intercept parameters of item and factors were added to the model (see Model 2b and 3b in Table 6). In model 2b, which tested the invariance of intercepts of observed variables, the SBχ2 difference test was insignificant and ΔCFI value was less than 0.01 (Model 2b: ΔSBχ2 = 6.81, df = 4, *p* < 0.001). These results suggests that there is no significant difference in the intercepts of the observed variables. But Model 3b, which examined the invariance of the intercepts of the hypothesized factors, indicated a significant difference test of SBχ2 (ΔSBχ2 = 44.48, df = 9, *p* < 0.001). However, ΔCFI value was again less than 0.01, leading us to conclude that Model 3b represents an adequate level of invariance. Similarly, these results suggest that there is no significant difference in the intercepts of the hypothesized factors between BA and MA teachers.

### Testing Group Differences in the Hypothesized Factor Means

In order to estimate the d between the hypothesized factor means across gender, the females were considered as a reference group and their latent means were equalized to zero. Then the latent means of the male group indicate the distinction in factor means between the two groups. The analysis demonstrated that there were substantially significant mean differences between the groups (i.e., male & female) on the critical and affective factors.

The result of Wald *z* test indicated that the female teachers obtained lower scores on the critical reflection (difference = 0.36, *z* = 3.19, *p* = 0.024) and higher scores on the affective factor (difference = −0.28, *z* = −2.16, *p* = 0.041) than the male teachers. The gender difference in practical (difference = 0.07, *z* = 0.532, *p* = 0.411), cognitive (difference = −0.04, *z* = −0.482, *p* = 0.452), meta-cognitive (difference = 0.03, *z* = 0.212, *p* = 0.274) and moral (difference = 0.09, *z* = 0.756, *p* = 0.641) factors was not statistically significant.

To estimate the latent means differences across different educational degrees, the BA group was selected as a reference or baseline group and its latent mean was set to zero. Then the latent mean of the MA group was estimated. Results of the Z statistic indicated that MA teachers obtained higher scores on the critical (difference = 0.42, *z* = 4.69, *p* = 0.016) and cognitive factors (difference = 0.26, *z* = 2.15, *p* = 0.034). The educational degree difference between BA and MA teachers in practical (difference = 0.08, *z* = 0.632, *p* = 0.426), affective (difference = −0.05, *z* = −0.491, *p* = 0.562), meta-cognitive (difference = −0.03, *z* = 0.314, *p* = 0.204) and moral (difference = 0.07, *z* = 0.584, *p* = 0.536) factors was not statistically significant.

## Discussion

To the best knowledge of the researchers, this research is the first independent empirical evidence to investigate the psychometric properties of the widely used L2 reflective inventory. For this purpose, the findings documented evidence regarding the construct validity and measurement invariance of the scale. In so doing, a large-scale representative dataset of practicing Iranian EFL teachers were recruited. The results of the confirmatory factor analyses supported the multidimensionality of the adapted version of the teacher reflection scale and revealed that teacher reflection was a multidimensional construct encompassing practical, cognitive, meta-cognitive, affective, critical and moral factors. Overall, the six-factor model showed an overall good fit. This finding supports the appropriateness and adequacy of the initial six-component model introduced by Akbari et al. (2010). The goodness of the fit indices for the new scale of L2 reflection with morality as one of its components verified the previous literature on the importance of morality as an important component of L2 reflective teaching (Valli, 1990; Hansen, 1998; Farrell, 2015, 2017). In addition, the findings of the study came up with empirical evidence supporting the measurement invariance of the underlying reflection factors across gender/educational degree groups.

Using covariance and mean structures, we examined the invariance of factor structure, factor loadings, intercepts, and mean differences across the heterogeneous groups of teachers. More technically, we tested the measurement invariance at two levels of metric invariance and scalar invariance. The results revealed invariant factor loadings of the six-factor model across gender as well as educational degree. These findings as obtained from the comparisons of the nested models according to the forward approach revealed that the adapted teacher reflection instrument measured similar constructs for both male/female and BA/MA teachers. Metric invariance is considered as an essential requirement in order to compare multiple groups (Meredith, 1993; Meredith and Teresi, 2006). The adapted version of reflection scale did also show the requirements of scalar invariance for gender and educational degree. Therefore, the outcomes revealed that the six-factor L2 teacher reflection model as operationalized by the adapted scale showed strong measurement invariance, showing both metric and scalar invariance (Meredith and Teresi, 2006; Dimitrov, 2010). Evidence of scalar invariance reveals that the factors in the adapted reflection scale are assessed on the same scales for both male/female and BA/MA teachers and that particular reflection scores have the same exact meaning for the teachers across both groups. The results for both metric and scalar invariance verify that the appearance of any distinction in the scores of the factors is likely to show potential group dissimilarities in the amount of teacher reflection rather than bias or systematic measurement error. Therefore, after confirming the invariance of factor loadings as well as intercepts, the differences in the latent means on the underlying six factors of reflection were examined.

Concerning group differences in the factor means, the findings revealed that the mean scores for the male EFL teachers were higher than those for the female ones on the critical factor, whereas the female teachers obtained higher scores on the affective factor. In other words, this might reveal that the male EFL teachers are more aware of the socio-political aspects of their pedagogy and more think about the social and political significance of their practice. On the other hand, the female teachers displayed more willingness to reflect on their students, their way of learning, and their emotional behavior and reactions in the classroom.

Additionally, the findings also suggested that the mean scores of MA teachers were higher on the critical and cognitive factors. More specifically, MA EFL teachers were not only more interested in viewing their practice as a socio-political activity but they were also more enthusiastic about their own professional growth by doing action research, taking part in conferences and workshops, and knowing about the professional literature of ELT enterprise. Given that the key variable distinguishing the two groups might be the degree of exposure to specialized ELT-related programs (or lack thereof) during their academic education, the MA teachers’ higher scores on the critical and cognitive factors might be attributed to the likely cause of such ELT-related programs. However, future research, both quantitative and qualitative, should verify the existence of these group differences on reflection components and also explore the reason for such between-group differences.

## Conclusion

The present study verified the multidimensional factor structure of L2 teacher reflection by investigating the psychometric properties and the measurement invariance of a slightly adapted teacher reflection scale. Unlike most of validation studies of assessment instruments in L2 research which only deal with the model fit, this study also investigated the measurement invariance as further evidence in construct validation. Testing for model fit pertains to the structural aspect of validity but does not address the generalizability aspect of validity (Dimitrov, 2010). The current study, however, investigated the structural and generalizability aspects of the unified conception of construct validity (Messick, 1995) through the examination of both the model fit and measurement invariance respectively. Moreover, unlike the original scale, the adapted scale includes the moral reflection as its underlying component which is compatible with the existing literature supporting the role of moral element of reflection (Valli, 1990; Hansen, 1998; Farrell, 2015). Additionally, the significant factor mean differences in reflection components across gender and educational degree groups might give rise to more qualitative and quantitative studies investigating such between-group differences in reflection components.

The findings of this study provided empirical support to ELT theoreticians and practitioners for assessing teacher reflection as an effective teacher-related variable. As far as teacher education program is concerned, the application of the adapted scale by teacher educators might be useful in assessing teacher reflection across its six underlying dimensions. The investigation of the mean differences for the dimensions of teacher reflection helps teacher educators to identify the practicing teachers who might not possess the adequate level of reflection in various dimensions. Consequently, interventions or remedies can be sought to be applied in order to prepare more reflective teachers by reflective practicums.

Although the findings of the present study are more appropriate for generalization to the Iranian teachers, this study may be of much significance from research point of view because the evaluation of measurement invariance across populations has been quite neglected in L2 research. However, further research is required to more fully investigate the psychometric properties and appropriateness of this adapted version of ELTRI using samples from different age groups, genders, teaching experience, and educational degrees across various environments (i.e., language institutes or public schools).

One major limitation of this study was that since the teacher participants of the present study were all from Iran and culturally non-diverse, the findings should be evaluated in more diverse populations. Additionally, future research should investigate whether the various dimensions of teacher reflection have different origins and consequences.

## Data Availability Statement

The original contributions presented in the study are included in the article/supplementary material, further inquiries can be directed to the corresponding author.

## Author Contributions

All authors have contributed equally to data collection, data analysis, research questions, topic development, writing the manuscript as well as its revision, and language editing.

## Conflict of Interest

The authors declare that the research was conducted in the absence of any commercial or financial relationships that could be construed as a potential conflict of interest.

## Publisher’s Note

All claims expressed in this article are solely those of the authors and do not necessarily represent those of their affiliated organizations, or those of the publisher, the editors and the reviewers. Any product that may be evaluated in this article, or claim that may be made by its manufacturer, is not guaranteed or endorsed by the publisher.
